# Evaluation of the Antifungal Efficacy of Natural Gum Acacia and Aloe Vera against *Candida albicans* on Conventional and Digital Denture Base Materials: An *In Vitro* Study

**DOI:** 10.1055/s-0045-1810440

**Published:** 2025-08-21

**Authors:** Mai Salah El-Din, Hend Zeitoun, Mostafa I. Fayad, Muhammad Sohail Zafar, Redhwan Saleh Al-Gabri, Ahmed Yaseen Alqutaibi, Rania Moussa

**Affiliations:** 1 Department of Prosthodontics, Alexandria University Main Hospital, Alexandria, Egypt; 2 Department of Microbiology and Immunology, Faculty of Pharmacy, Alexandria University, Alexandria, Egypt; 3 Department of Substitutive Dental Sciences, College of Dentistry, Taibah University, Al-Madinah Al-Munawwarah, Saudi Arabia; 4 Department of Clinical Sciences, College of Dentistry, Ajman University, Ajman, United Arab Emirates; 5 Center of Medical and Bio-allied Health Sciences Research, Ajman University, Ajman, United Arab Emirates; 6 School of Dentistry, University of Jordan, Amman, Jordan; 7 Department of Prosthodontics, Faculty of Dentistry, Ibb University, Ibb, Yemen

**Keywords:** gum acacia, aloe vera, CAD/CAM, PMMA, *Candida albicans*, denture base, denture disinfectant

## Abstract

**Objectives:**

This study evaluated the efficacy of Acacia Arabica and aloe vera solutions versus commercial effervescent tablets on *Candida* viability and adhesion to various denture bases with different materials and manufacturing techniques.

**Materials and Methods:**

A total of 96 square-shaped denture base specimens (24 per group) were fabricated from heat-cured polymethyl methacrylate (PMMA), thermoformed polyamide (FlexiUltra), milled computer-aided design and computer-aided manufacturing (CAD/CAM; Avadent), and 3D-printed (FormLabs) resins. Specimens were allocated into a control group (distilled water) and three experimental groups ( *n* = 6 per group) based on the immersion solutions: Gum acacia (GA), aloe vera, and alkaline peroxide denture cleansers. One specimen from each material subgroup was prepared for qualitative assessment. All specimens were incubated with *Candida albicans* for 48 hours and immersed in the designated disinfectant solution for 8 hours. Anti- *C. albicans* biofilm activity was assessed quantitatively using an MTT assay and qualitatively using a confocal laser scanning microscope (CLSM).

**Statistical Analysis:**

One-way analysis of variance (ANOVA) was employed for mean comparison, and data were considered significant at *p* < 0.05.

**Results:**

The reduction in mean *Candida* viability was greatest in the alkaline peroxide tablets across all denture base materials, followed by GA and aloe vera ( *p* < 0.001). GA demonstrated a statistically significant reduction in *Candida* levels in the thermoplastic polyamide ( *p* < 0.001). The mean viability of *Candida* in both alkaline peroxide tablets and GA was statistically similar in digital denture bases ( *p* > 0.05).

**Conclusions:**

The antifungal efficacy of alkaline peroxide denture cleansers was determined to be the highest. The cost-effective GA may serve as a viable denture disinfectant, particularly for thermoformed polyamide denture bases.

## Introduction

Removable dentures restore lost teeth, improve oral function, and enhance the quality of life, especially in low-income countries. However, they pose clinical challenges like denture stomatitis (DS), 1 a prevalent inflammatory condition affecting the denture-supporting oral mucosa. DS is often linked to poor oral hygiene, reduced salivary flow, irritation, and microbial infections. 2 The primary fungal culprit in DS is *Candida albicans* ( *C. albicans* ), which colonizes denture surfaces either alone or in conjunction with other microbes. It is found in 45 to 65% of healthy individuals and 60 to 100% of denture wearers. 2 3

Good oral hygiene and strict denture care are essential to prevent and manage *Candida* complications associated with dentures. 4 Common denture hygiene practices include mechanical brushing (with or without toothpaste), denture cleansing solutions, and ultrasonic cleaning. 5 6 The careful selection of denture cleaning methods is crucial for preserving the physical properties of the denture base material. Research indicates that brushing with toothpaste can roughen denture surfaces and increase plaque accumulation, whereas brushing without toothpaste may not effectively eliminate microorganisms. 7

The ability of *C. albicans* to form protective biofilms makes it particularly virulent, shielding it from antifungal treatments and promoting its growth. DS is typically treated with topical or systemic antifungals like nystatin, miconazole, and fluconazole. While effective, these treatments face limitations such as potential drug resistance, side effects (e.g., gastrointestinal disturbances, allergic reactions), and the need for repeated or prolonged administration. 8

Medicinal plant extracts and herbal medicine are increasingly recognized as safe, reliable alternatives to conventional antimicrobials. Plant-based compounds show promise for denture-related oral health. Researchers are exploring natural antifungals like curcumin, cinnamaldehyde, and eugenol to inhibit *Candida* biofilms. These agents suppress *Candida* growth and hinder biofilm formation by disrupting fungal cellular processes, including cell cycle progression, mitochondrial activity, and the biosynthesis of ergosterol, chitin, and glucan. 9

Acacia arabica bark, a medicinal plant extensively utilized in traditional Indian and African medicine, commonly referred to as the GA tree, exhibits antimicrobial and antibiofilm properties. GA is rich in bioactive compounds, including tannins and flavonoids, which have demonstrated potent antimicrobial activity against oral pathogenic microorganisms. 9 10

Aloe vera ( *Aloe barbadensis miller* ) from the Liliaceae family, has a long history in traditional medicine and is widely used in skincare, cosmetics, medicine, health care, and food. 11 12 This plant contains over 200 biologically active substances, including hormones, enzymes, anthraquinones, and amino acids. Aloe vera offers numerous therapeutic benefits, such as wound healing, antibacterial, anti-inflammatory, antioxidant, and moisturizing effects. 13

Polymethyl methacrylate (PMMA) remains the predominant material for the fabrication of removable dentures. 14 Thermoformed acrylic resins are monomer-free, providing enhanced retention and improved load distribution compared to conventional heat-cured PMMA. 15 The advent of digital dentures, fabricated through computer-aided design and computer-aided manufacturing (CAD/CAM) technologies, has revolutionized denture production and improved patient care. 16 Digitally fabricated dentures are either milled (subtractive) or 3D-printed (additive). While milling is common, 17 3D printing offers advantages such as cost-effectiveness and the simultaneous production of multiple dentures. 18 Consequently, the aim of this study was to evaluate and compare the efficacy of gum acacia, aloe vera, and alkaline peroxide denture disinfectant agents against *C. albicans* biofilm on conventional, thermoformed, milled, and 3D-printed denture base resins. The null hypothesis postulates that there will be no significant differences in the impact of GA, aloe vera, and alkaline peroxide tablets on the viability and adherence of *C. albicans* to the tested denture base materials.

## Materials and Methods

### Sample Size Calculation

The estimated sample size was determined based on a significance level of 5% and a statistical power of 80% (α = 0.05). The total required sample size for testing was calculated to be 96 specimens ( *n* = 24 per group), which included one specimen from each subgroup for imaging purposes. The sample size was estimated using G*Power Version 3.1.9.7. 19

### Study Design

Square specimens measuring 10 mm × 10 mm × 3 mm were fabricated from four different denture base resins: Conventional PMMA (group I), thermoformed polyamide (group II), CAD/CAM-milled resin (group III), and 3D-printed resin (group IV). The 96 specimens from each group were further subdivided according to the immersion solutions into four subgroups ( *n* = 6 each): Control distilled water (C-group), GA 50% wt/v suspension (GA group), aloe vera 30% wt/v solution (AV group), and effervescent alkaline peroxide solution (CR group).

### Specimens' Preparation

The trade names and the composition of the denture base materials used in this study are presented in Table 1 .

**Table 1:** Composition and description of denture base materials used in the study

Group	Material Type	Trade Name	Manufacturer	Composition	Fabrication technique
I	Heat-cured PMMA	*Acrostone* ®	Acrostone Dental & Medical Supplies, Egypt	Polymethyl methacrylate, benzoyl peroxide	Conventional water-bath curing
II	Thermoformed polyamide	*Flexi Ultra* ®	Flexafil S.A.C.I., Argentina	Polyamide (nylon-based thermoplastic resin)	Injection molding
III	CAD/CAM milled resin	*AvaDent* ®	Global Dental Science, Netherlands	Pre-polymerized PMMA blocks	Subtractive milling
IV	3D-printed light-cured resin	*FormLabs Denture Base* ®	FormLabs Inc.	Photopolymerizable methacrylate-based resin	Additive 3D printing (SLA)

Abbreviations: CAD/CAM, computer-aided design and computer-aided manufacturing; PMMA, polymethyl methacrylate.

Group I: Heat-cured PMMA ( *Acrostone* ® heat-cured denture base, Acrostone Dental & Medical Supplies, Egypt; 2023) was utilized. Wax patterns of the specified dimensions were prepared using a prefabricated metal die measuring 10 mm × 10 mm × 3 mm. PMMA specimens were fabricated through the conventional method of polymerization in a hot water bath. 20
Group II: Thermoformed polyamide ( *Flexi Ultra* ®, Flexafil S.A.C.I. Leopoldo Marechal, Argentina “orange pink 78”). Specimens were produced using an injection molding technique, adhering to the manufacturer's instructions under pressure (5–7 bars) and heat (280 °C) for 15 minutes. 15
Group III: CAD/CAM-milled prepolymerized resin blocks ( *AvaDent* ® denture base puck, AvaDent, Global Dental Science Europe, Tilburg, The Netherlands; shade: light pink) were sectioned to the specified dimensions using a diamond saw (IsoMet 5000 Linear Precision Saw, Buehler) in a wet environment. 19
Group IV: 3D-printed resin specimens ( *FormLabs Denture Base* ®, Somerville, MA; shade: light pink) were designed using Open Software (123D Design, Autodesk, version 2.2.14, CA). The specimens were printed at a 90-degree orientation, employing sequential photopolymerization with a layer thickness of 50 μm. Following the manufacturer's guidelines, the specimens underwent postcuring in the appropriate light-curing unit. 19

One trained laboratory technician finished all the denture base material specimens using progressively finer grades of silicon carbide bur for 2 minutes, followed by 150 grit sandpaper for an additional 2 minutes (MicroCut PSA; Buehler, IL). Specimen surfaces were prepolished using a rubber bur and subsequently treated with fine pumice on a wet rag wheel. 21

To eliminate any microbiological contamination, the specimens were submerged in 70% ethanol for 30 minutes. Subsequently, to remove any residual ethanol, they were rinsed with sterile saline and immersed in sterile distilled water for 48 hours at 37 °C to mitigate the effects of potential residual monomer release. 22

### Immersion Solutions

Control subgroup: The specimens were immersed in sterile distilled water.

Subgroup (GA) suspension: Natural gum acacia, free from additives, was procured locally. The entire gum was thoroughly washed with clean water to remove any contaminants. The cleaned gum was spread on a clean, dry sheet and air-dried in the shade. Once completely dry and brittle, it was ground into a fine powder. 23 The extract was subsequently mixed with sterile water at a concentration of 50% wt/v. 19

Subgroup (aloe vera) solution: Aloe vera leaves were obtained locally, washed with tap water, and then rinsed with distilled water. The leaves were dissected longitudinally using a sterile knife, and the gel was carefully scooped out with a sterile sharp spatula to avoid contamination with plant fibers. 24 The aloe vera gel was thoroughly macerated to yield a fine, homogeneous paste. About 30 g of this paste was then mixed with 100 mL water, yielding a 30% wt/v suspension. 24

Subgroup (effervescent alkaline peroxide tablets): The solution was prepared following the manufacturer's guidelines by adding one effervescent alkaline peroxide tablet (Corega Tabs, Block Drug Company, Inc., Jersey City, NJ) to 200 mL of warm sterile water. The tablet comprises potassium monopersulfate, sodium bicarbonate, sodium lauryl sulfoacetate, sodium perborate monohydrate, and sodium polyphosphate. 25

### Microbial Culture and Biofilm Formation

*Candida albicans* strain ATCC 10231 was obtained from the Microbiology Department at the Faculty of Pharmacy, Alexandria University, Alexandria, Egypt. A saline suspension of the strain was prepared and adjusted to achieve a turbidity equivalent to a 0.5 McFarland standard (approximately 10 ^6^ colony-forming units [CFUs]/mL). This suspension was subsequently utilized to inoculate Roswell Park Memorial Institute medium (RPMI-1640 medium, Biowest - The Serum Specialist, Rue de la Caille, 49340 Nuaillé, France). 26

The resin denture base specimens underwent sterilization for 20 minutes in an autoclave (GETINGE HS33-series, GETINGE IC Production Poland Sp. Z O O, ul. Szkolna 30, Plewiska, 62-064, Poland). Following sterilization, the specimens were individually placed at the bottom of sterile 24-well cell culture plates (SPL Life Sciences Co., Ltd., 48, Geumgang-ro 2047 beon-gil, Naechon-Myeon, Pocheon-si, Gyeonggi-do, Korea). An aliquot of 1.5 mL of the prepared *C. albicans* suspension in RPMI medium was added to each well containing the denture base specimens. The plates were incubated aerobically at 37 °C for 48 hours to facilitate fungal growth and adhesion to the specimens. An uninoculated well containing sterile RPMI medium served as the negative control (blank well). 26

Upon completion of the incubation period, the denture base specimens were washed three times with 1.5 mL of phosphate-buffered saline (PBS) to remove loosely adherent *Candida* cells, and they were subsequently transferred to a new 24-well cell culture plate. 20

The immersion solutions were freshly prepared, including distilled water (control group), a 50% GA suspension, a 30% AV suspension, and an alkaline peroxide tablet (CR) solution. About 2 mL of the immersion solution was added to each specimen in the test groups, and the specimens were immersed for 8 hours. 20 After the immersion period, the specimens were washed three times with PBS to remove any residual immersion solution and then analyzed for *C. albicans* adhesion and biofilm formation. 19

### Biofilm Assessment by MTT Assay

The percentage of biofilm formation by *C. albicans* was assessed using a colorimetric MTT assay. The MTT reagent (3-(4,5-dimethylthiazol-2-yl)-2,5-diphenyltetrazolium bromide) was prepared in PBS at a concentration of 0.5 mg/mL. About 100 μL of the reagent was added to each test and control well. The specimens were subsequently incubated at 37 °C for 3 hours in the dark, until the initial signs of color change were observed. Following incubation, the medium was aspirated, and the formed formazan crystals were solubilized by the addition of 50 μL of dimethyl sulfoxide (DMSO) per well, followed by a 30-minute incubation at 37 °C in the dark. The intensity of the dissolved formazan crystals (indicated by a purple color) was quantified using an ELISA plate reader at a wavelength of 540 nm. The MTT assay was conducted in triplicate, and the average value was calculated. 27 The MTT assay operates on the principle of enzymatic reduction of the MTT reagent by mitochondrial enzymes present in viable cells, producing purple formazan crystals. These formazan crystals are subsequently dissolved in a solvent such as DMSO, and the resulting solution is measured spectrophotometrically. The absorbance values obtained reflect the quantity of formazan produced, which correlates with the number of viable cells. A higher concentration of formazan crystals and a more intense purple color indicate increased cellular metabolic activity.

### Cell Viability Calculation

To accurately quantify the effect of the immersion solutions on *C. albicans* viability, absorbance values obtained from the MTT assay were corrected and analyzed as follows:

First, the absorbance reading of each specimen was corrected by subtracting the absorbance of the blank well (containing only medium and MTT reagent, without cells). This correction eliminated background signals from non-specific absorbance by the culture medium, MTT reagent, or other experimental components unrelated to cellular metabolic activity. The resulting value more accurately reflected the formazan produced exclusively by viable *C. albicans* cells. 28

Relative cell viability was then calculated by comparing the corrected absorbance of each test group to that of the untreated control group (distilled water), using the following formula 28 :

*Mean sample* = mean absorbance of specimens treated with an immersion solution.
*Mean blank* = absorbance of blank wells.
*Mean control* = mean absorbance of the control specimens.

This calculation enabled the quantitative assessment of the reduction in metabolic activity, and thus viability, of *C. albicans* in each experimental group compared to the control. Lower percentages indicate greater antifungal efficacy of the tested immersion solution, as reflected by a greater reduction in viable *C. albicans* cells.

### *Candida albicans'* Adherence Evaluation by Confocal Laser Scanning Microscope

To qualitatively evaluate *C. albicans* adherence to the denture base resin specimens, inverted CLSM (Leica DMi8, Leica Microsystems, GmbH, Germany) was employed at the Centre of Excellence for Research in Regenerative Medicine and its Applications (CERRMA), Faculty of Medicine, Alexandria University, Alexandria, Egypt. Representative denture base specimens were washed once with 1.5 mL of PBS and subsequently fixed in 2.5% glutaraldehyde in 0.1 M phosphate buffer (pH 7.2) at 4 °C for 1 hour. Following another wash in the buffer, specimens were postfixed in 1% osmium tetroxide in the same buffer for 30 minutes. The specimens were then dehydrated through a graded ethanol series (30%, 50%, 70%, 90%, and 100%) and were critical point-dried in CO _2_ using a Polaron Critical Point Dryer. Subsequently, the specimens were sputter-coated with an approximately 100-nm layer of gold (SCD 050 Sputter Coater, Baltic, Liechtenstein). 19 This gold coating was applied to enhance the scattering of laser light and to provide adequate contrast for imaging and visibility of fungal cells. 29 The specimens were positioned on the CLSM and imaged at a magnification of 20 × . A series of two-dimensional optical views parallel to the surface were captured from various regions of interest on each specimen, facilitating direct visualization of fungal aggregates, individual fungal cells, and areas exhibiting dense *Candida* colonization. 29

### Statistical Analysis

The data analysis was conducted using the Statistical Package for the Social Sciences (SPSS; v. 27, IBM SPSS Inc., Armonk, NY). Descriptive statistics, including means and standard deviations, were calculated. The normality of the data distribution was assessed using the Shapiro–Wilk test. A one-way analysis of variance (ANOVA) was employed to compare means, followed by Tukey's post hoc test for pairwise comparisons. Differences were deemed statistically significant at *p* < 0.05.

## Results

**Fig. 1:**
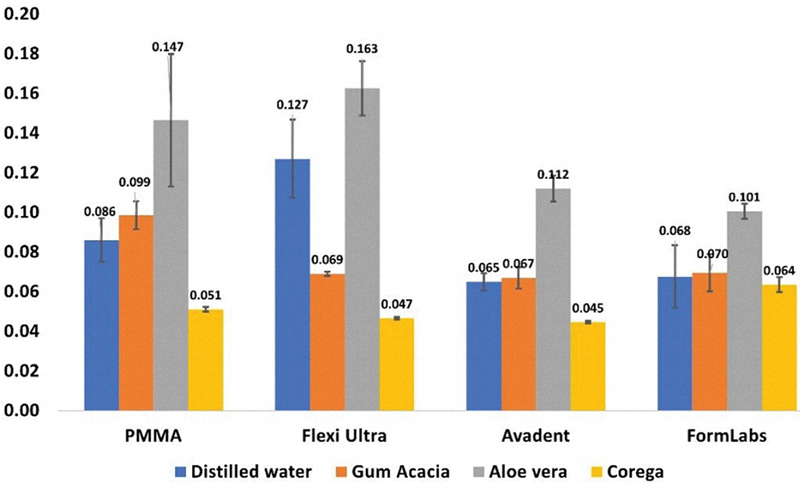
MTT assay results of the mean absolute absorbance of the reagent reflecting the viability of *Candida albicans* cells adhered to conventional, thermoplastic polyamide, milled, and 3D-printed denture base materials after immersion in disinfectant solutions. MTT assay, 3-(4,5-dimethylthiazol-2-yl)-2,5-diphenyltetrazolium bromide assay; PMMA, polymethyl methacrylate.

The absolute absorbance values (mean ± standard deviation) from the MTT assay for the evaluated denture base materials, following immersion in various denture disinfectant solutions are presented in Fig. 1 .

In Group I, the CR subgroup exhibited the lowest viability of *Candida* cells compared to the control ( *p* = 0.015), while the AV subgroup exhibited the highest ( *p* < 0.001). No significant differences were found between the GA and control subgroups ( *p* = 0.630). Group II showed significant differences among all pairwise subgroups ( *p* < 0.001), with the CR subgroup exhibiting the lowest *Candida* viability, followed by the GA subgroup, and then the AV subgroup. In Group III, no significant difference was found between the control and GA subgroups ( *p* = 0.888). CR subgroup showed significantly lower *Candida* viability ( *p* < 0.001), and the AV subgroup showed higher viability ( *p* < 0.001). Group IV results indicated no significant difference between the control and the GA ( *p* = 0.983) or CR ( *p* = 0.887) subgroups, while the AV subgroup showed a significant increase in *Candida* viability ( *p* < 0.001).

**Fig. 2:**
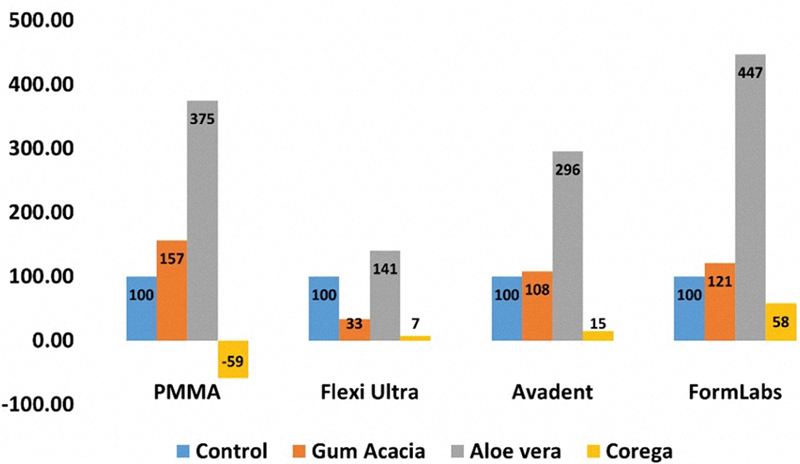
Comparison of MTT reagent relative absorbance of the tested materials subgroups compared to the control after the immersion in disinfectant solutions. MTT, 3-(4,5-dimethylthiazol-2-yl)-2,5-diphenyltetrazolium bromide; PMMA, polymethyl methacrylate.

The relative viability of the *C. albicans* compared to the control of the tested groups is presented in Fig. 2 . A significant difference in relative cell viability was observed across the denture base material subgroups ( *p* < 0.001).

**Fig. 3:**
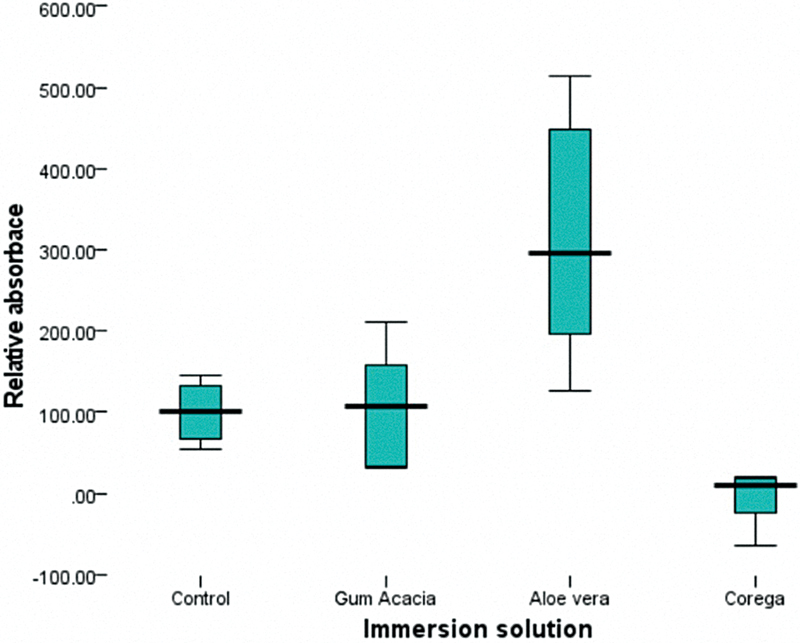
Box plot illustrating the distribution of mean collective relative absorbance of the MTT reagent as affected by the immersion solution performance relative to the control group.

The overall efficacy of the tested antifungal agents is presented in Fig. 3 . CR subgroups demonstrated the highest effectiveness, followed by the GA subgroups, while the AV subgroup exhibited the least effectiveness.

**Fig. 4:**
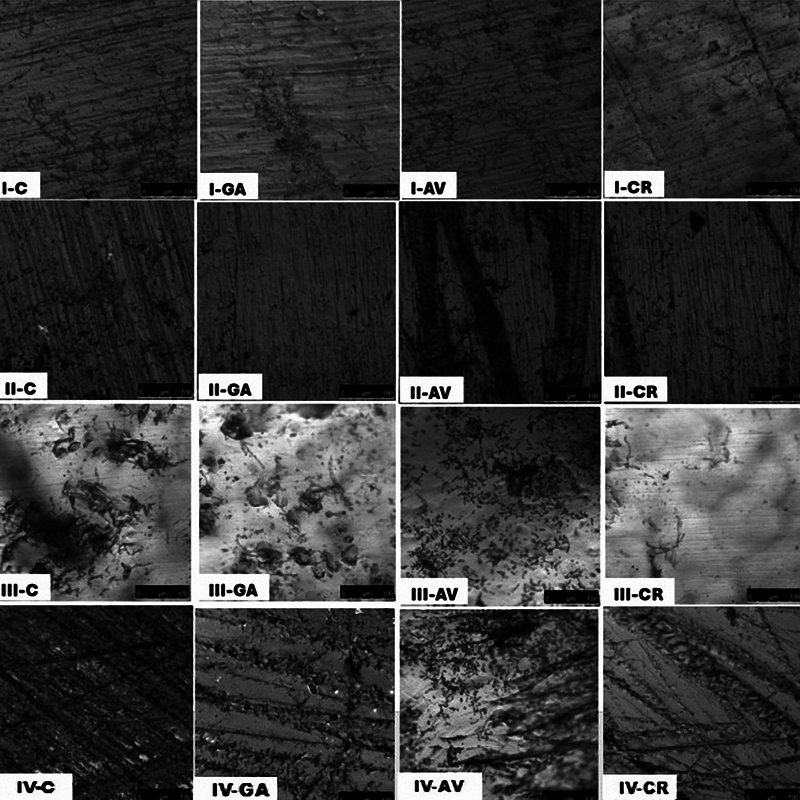
Confocal laser scanning microscopy images of representative specimens of the denture base materials subgroups showing the variation in clustering of the *Candida albicans* cells on the surface.

CLSM images ( Fig. 4 ) demonstrated varying levels of fungal adherence across the experimental groups. Dense clusters of fungal cells colonized the acrylic resin surface in subgroups I-C, I-AV, II-C, II-AV, III-AV, and IV-AV. Fewer and more sparsely distributed fungal cells were observed in subgroups I-CR, II-GA, II-CR, and III-CR.

## Discussion

This study assessed and compared the antifungal efficacy of gum acacia, aloe vera, and commercial alkaline peroxide tablets against *C. albicans* viability and adherence on four types of denture base materials. The results demonstrated significant differences among the tested disinfectants, leading to rejection of the null hypothesis.

The MTT colorimetric assay was employed in this study to assess *Candida* cell viability by measuring the metabolic activity of adherent fungal cells on denture base materials. According to ISO 10993-5:2010, a cell viability reduction exceeding 30% indicates a potent cytotoxic effect. 28 Among all tested disinfectants, alkaline peroxide effervescent tablets exhibited the highest anti- *C. albicans* biofilm efficacy. These tablets clean dentures by releasing carbon dioxide, which helps dislodge and remove debris, stains, and biofilms. Additionally, oxidizing agents such as potassium monopersulfate or sodium perborate generate oxygen, disrupting microbial cell walls. 30 However, prolonged use of alkaline peroxide tablets can deteriorate denture surface topography. Moussa et al., 25 reported that immersion in these tablets for 300 hours increased surface roughness beyond clinically acceptable levels (0.2 μm) for both conventionally and digitally fabricated denture base materials.

Aloe vera, although widely recognized for its medicinal properties, 31 was ineffective as an immersion disinfectant in this study. One possible explanation is that certain polysaccharides present in aloe vera, such as veracylglucan C, may actually promote the growth of *Candida* rather than inhibit it. Additionally, variations in the antifungal efficacy of aloe vera have been reported in the literature, which may be attributed to differences in plant origin, chemical composition, and extraction or isolation techniques. 31 32 Standardized methods for aloe vera gel production are necessary to prevent its degradation. Notably, our findings contrast with those of Abduljabbar et al., 33 who observed greater sensitivity of *C. albicans* isolated from nylon dentures to aloe vera compared to acrylic dentures, and with Shilpa et al., 24 who reported higher antifungal activity for aloe vera gel extract than for the leaf extract. These discrepancies further highlight the need for standardized preparation protocols to assess aloe vera's antifungal potential reliably.

Gum acacia showed a moderate antifungal effect, but significantly reduced *C. albicans* viability in the thermoplastic polyamide. This could be attributed to the interaction between its bioactive compounds (such as tannins and flavonoids) and the surface characteristics of polyamide, which is known for higher surface roughness. 25 This roughness could enhance gum acacia's adherence and antifungal action. 9 10

Interestingly, digitally fabricated denture bases (milled and 3D-printed) showed minimal differences in *C. albicans* viability and adherence between the disinfectant and control groups. This suggests that the smoother, less porous surfaces produced by digital manufacturing may inherently resist fungal colonization, reducing the relative impact of immersion disinfectants. 34

This *in vitro* study has several limitations. The controlled laboratory conditions do not fully replicate the complex oral environment, where factors such as saliva, other microorganisms, and host immune responses can influence *Candida* behavior. Additionally, variables like temperature changes, pH fluctuations, and mechanical stresses experienced during daily denture use were not simulated. Only selected denture base materials were tested, which may not represent all materials used clinically, and differences in properties such as porosity and hydrophobicity could affect the results. Finally, the relatively short study duration may not reflect the long-term effects of antifungal treatments. Therefore, *in vivo* studies are needed to confirm these findings under real-world conditions.

## Conclusion

Within the constraints of the present study, it concluded that alkaline peroxide tablets exhibited the highest antifungal efficacy across all denture base materials. Cost-effective gum acacia showed moderate antifungal activity, with its most pronounced effect observed on the thermoplastic polyamide resin. Aloe vera demonstrated the lowest antifungal efficacy, with cell viability values similar to or higher than those of the control group across all materials. While polyamide denture bases are less commonly used in clinical practice, the findings highlight material-specific interactions that may inform future research and formulation of disinfectant protocols.

## References

[1] Sakr H M AbdulSalam M R Fayad M I Moussa R Alzahrani A AH Microbial adhesion to different thermoplastic denture base materials in Kennedy Class I partially edentulous patients Cureus 2024 16 05 e60421 38756717 10.7759/cureus.60421 PMC11097705

[2] Sartawi S Y Abu-Hammad S Al-Omoush S Denture stomatitis revisited: A summary of systematic reviews in the past decade and two case reports of papillary hyperplasia of unusual locations Int J Dent 2021 2021 01 7.338143E6 10.1155/2021/7338143 PMC8528609 34691183

[3] Gendreau L Loewy Z G Epidemiology and etiology of denture stomatitis J Prosthodont 2011 20 04 251 260 21463383 10.1111/j.1532-849X.2011.00698.x

[4] Algabri R Alqutaibi A Y Altayyar S Behaviors, hygiene habits, and sources of care among removable complete and partial dentures wearers: a multicenter cross-sectional study Clin Exp Dent Res 2024 10 02 e867 38433293 10.1002/cre2.867 PMC10909811

[5] Schmutzler A Rauch A Nitschke I Lethaus B Hahnel S Cleaning of removable dental prostheses–a systematic review J Evid Based Dent Pract 2021 21 04 101644 34922732 10.1016/j.jebdp.2021.101644

[6] Chan R Zhang J McGrath C Tsang P Lam O Lam O A randomized trial of the effectiveness of an ultrasonic denture hygiene intervention program among community dwelling elders Eur Oral Res 2023 57 02 83 89 37525857 10.26650/eor.20231025114 PMC10387143

[7] Chang Y-H Lee C-Y Hsu M-S Du J-K Chen K-K Wu J-H Effect of toothbrush/dentifrice abrasion on weight variation, surface roughness, surface morphology and hardness of conventional and CAD/CAM denture base materials Dent Mater J 2021 40 01 220 227 33028789 10.4012/dmj.2019-226

[8] Emami E Kabawat M Rompre P H Feine J S Linking evidence to treatment for denture stomatitis: a meta-analysis of randomized controlled trials J Dent 2014 42 02 99 106 24316341 10.1016/j.jdent.2013.11.021

[9] Shariati A Didehdar M Razavi S Heidary M Soroush F Chegini Z Natural compounds: a hopeful promise as an antibiofilm agent against *Candida* species Front Pharmacol 2022 13 917787 35899117 10.3389/fphar.2022.917787 PMC9309813

[10] Ramalingam K Amaechi B T Antimicrobial effect of herbal extract of *Acacia arabica* with triphala on the biofilm forming cariogenic microorganisms J Ayurveda Integr Med 2020 11 03 322 328 30389224 10.1016/j.jaim.2018.01.005 PMC7527819

[11] Kaur S Bains K *Aloe barbadensis miller* (aloe vera) Int J Vitam Nutr Res 2023 94 308 321 37915246 10.1024/0300-9831/a000797

[12] Sánchez M González-Burgos E Iglesias I Gómez-Serranillos M P Pharmacological update properties of aloe vera and its major active constituents Molecules 2020 25 06 1324 32183224 10.3390/molecules25061324 PMC7144722

[13] Memon M R Memon H Shoro M Effectiveness of chitosan versus natural aloe vera on *Candida* adherence in denture soft lining material Scientifica (Cairo) 2024 2024 01 9.918914E6 10.1155/2024/9918914 PMC10789509 38225940

[14] Alqutaibi A Y Baik A Almuzaini S A Polymeric denture base materials: a review Polymers (Basel) 2023 15 15 3258 37571151 10.3390/polym15153258 PMC10422349

[15] Golmohammadi F Ghasemi Z Comparative study of the compressive strength of heat-cure acrylic resin with flexible thermoplastic acrylic Contemporary Orofacial Science 2024 2 02 26 32

[16] Steinmassl O Dumfahrt H Grunert I Steinmassl P-A CAD/CAM produces dentures with improved fit Clin Oral Investig 2018 22 08 2829 2835 10.1007/s00784-018-2369-2 29468600

[17] Kattadiyil M T AlHelal A An update on computer-engineered complete dentures: a systematic review on clinical outcomes J Prosthet Dent 2017 117 04 478 485 27881317 10.1016/j.prosdent.2016.08.017

[18] Abduo J Lyons K Bennamoun M Trends in computer-aided manufacturing in prosthodontics: a review of the available streams Int J Dent 2014 2014 01 783948 24817888 10.1155/2014/783948 PMC4000974

[19] El-Din M S Shehat M G Kamal S M Potential antifungal effect of acacia arabica extract versus sterile water regarding surface topography of denture base materials: an in-vitro study Alex Dent J 2024 50(2B):116 122

[20] Khurana P Singhal R Agarwal S K Kalpana K Comparative evaluation of the effect of two plant extract and denture cleanser on the staining and anti-fungal efficacy of denture base resin: an in vitro study J Dent Oral Biol 2022 7 03 1 7

[21] El-Din M S Badr A M Agamy E M Mohamed G F Effect of two polishing techniques on surface roughness of three different denture base materials (an in vitro study) Alex Dent J 2018 43 03 34 40

[22] Kurt A Erkose-Genc G Uzun M Sarı T Isik-Ozkol G The effect of cleaning solutions on a denture base material: elimination of candida albicans and alteration of physical properties J Prosthodont 2018 27 06 577 583 27599151 10.1111/jopr.12539

[23] Alsadon O Alkhureif A A Khan A A Effect of gum arabic powder on the mechanical properties of denture base acrylic Pak J Med Sci 2023 39 01 223 226 36694769 10.12669/pjms.39.1.6937 PMC9842989

[24] Shilpa M Bhat V Shetty A V Reddy M S Punde P Antifungal activity of aloe vera leaf and gel extracts against *Candida albicans* : an in vitro study World J Dentist 2020 11 01 37

[25] Moussa R Ellakany P Fouda S M El-Din M S Comparative evaluation of the effects of laser and chemical denture disinfectants on the surface characteristics of CAD-CAM and conventional denture resins: an in vitro experimental study J Prosthodont 2024(e-pub ahead of print) 10.1111/jopr.13952 PMC13456428 39300670

[26] Osman R B Khoder G Fayed B Kedia R A Elkareimi Y Alharbi N Influence of fabrication technique on adhesion and biofilm formation of *Candida albicans* to conventional, milled, and 3D-printed denture base resin materials: a comparative in vitro study Polymers (Basel) 2023 15 08 1836 37111983 10.3390/polym15081836 PMC10146129

[27] Bahuguna A Khan I Bajpai V K Kang S CMTT assay to evaluate the cytotoxic potential of a drug. Bangladesh J Pharmacol 2017;12(2): Online: Apr 8–2017

[28] Novotná B Holík P Morozova Y Rosa M Galandáková A Langová K Evaluation of cytotoxicity of the dental materials TheraCal LC, TheraCal PT, ApaCal ART and Biodentine used in vital pulp therapy: *In vitro* study Dent J 2024 12 08 249 10.3390/dj12080249 PMC11352889 39195093

[29] Rashid H Application of confocal laser scanning microscopy in dentistry J Adv Microsc Res 2014 9 04 245 252

[30] Peracini A Regis R R Souza R F Pagnano V O Silva C H Paranhos H F Alkaline peroxides versus sodium hypochlorite for removing denture biofilm: a crossover randomized trial Braz Dent J 2016 27 06 700 704 27982182 10.1590/0103-6440201600913

[31] Hamman J H Composition and applications of aloe vera leaf gel Molecules 2008 13 08 1599 1616 18794775 10.3390/molecules13081599 PMC6245421

[32] Hęś M Dziedzic K Górecka D Jędrusek-Golińska A Gujska E Aloe vera (L.) Webb.: natural sources of antioxidants–a review Plant Foods Hum Nutr 2019 74 03 255 265 31209704 10.1007/s11130-019-00747-5 PMC6684795

[33] Abduljabbar M A Ali E Kamil N B Al-Kahayyat F Effect of olea extracts on oral candida in patients wearing dentures of different base materials Dental Sci 2016 1 01 1 15

[34] Freitas R FCP Duarte S Feitosa S Physical, mechanical, and anti-biofilm formation properties of CAD-CAM milled or 3D printed denture base resins: *In vitro* analysis J Prosthodont 2023 32(S1):38 44 35661475 10.1111/jopr.13554

